# Asparaginyl endopeptidase promotes the invasion and metastasis of gastric cancer through modulating epithelial-to-mesenchymal transition

**DOI:** 10.18632/oncotarget.8879

**Published:** 2016-04-20

**Authors:** Yuehong Cui, Yan Wang, Hong Li, Qian Li, Yiyi Yu, Xiaojing Xu, Bei Xu, Tianshu Liu

**Affiliations:** ^1^ Medical Oncology Department, Zhongshan Hospital, Fudan University, Shanghai, China

**Keywords:** asparaginyl endopeptidase, metastasis, epithelial-to-mesenchymal transition, signaling pathway, gastric cancer

## Abstract

Asparaginyl endopeptidase (AEP) is a lysosomal protease often overexpressed in gastric cancer. AEP was expressed higher in peritoneal metastatic loci than in primary gastric cancer. Then we overexpressed AEP or knocked it down with a lentiviral vector in gastric cancer cell lines and detected the cell cycle arrest and the changes of the invasive and metastatic ability *in vitro* and *in vivo*. When AEP was knocked-down, the proliferative, invasive and metastatic capacity of gastric cancer cells were inhibited, and the population of sub-G1 cells increased. AEP knockdown led to significant decrease of expression of transcription factor Twist and the mesenchymal markers N-cadherin, ß-catenin and Vimentin and to increased expression of epithelial marker E-cadherin. These results showed that AEP could promote invasion and metastasis by modulating EMT. We used phosphorylation-specific antibody microarrays to investigate the mechanism how AEP promotes gastric cancer invasion and metastasis, and found that the phosphorylation level of AKT and MAPK signaling pathways was decreased significantly if AEP was knocked-down. Therefore, AKT and MAPK signaling pathways took part in the modulation.

## INTRODUCTION

The incidence of gastric cancer is higher in China than most of other countries. By some estimates, approximately 300,000 people will die of gastric cancer annually in China [1]. Peritoneal metastasis is one of the most common metastases in gastric cancer, and 40-50% of patients are likely to develop a peritoneal metastasis after receiving radical resection, which is very difficult to treat [2]. Although the “seed-soil” hypothesis has often been used to explain the mechanism of peritoneal metastasis in the past, the metastatic process is very complex and involves many genes without a single “driver gene”. Based on histological and biological behavior, Lauren (1965) divided gastric cancer into the intestinal type and diffuse type. It has been reported that multivariate prognostic analysis revealed that diffuse type histology was an independent prognostic factor in gastric cancer [3], and diffuse type gastric cancer was associated with deeper invasion and cytology test positive, then led to more dismal prognosis [4–5]. In 2013, Patrick Tan's team classified gastric cancer into mesenchymal, proliferative and metabolic types [6]. The mesenchymal type was found to be similar to the diffuse type and also displayed a high mutant frequency of the CDH1 gene, which encodes E-cadherin. Notably, E-cadherin is an important marker of epithelial-to-mesenchymal transition (EMT).

Asparaginyl endopeptidase (AEP, also called legumain) is a lysosomal protease and a unique member of the C13 family peptidases with strict specificity for asparagine bond cleavage [7–8]. AEP has been identified in the lysosome as well as the extracellular matrix and surface of tumor cells. AEP overexpression was observed in some solid tumors, such as breast cancer, colon cancer, lung cancer, and acute lymphoblastic leukemia [9–14]. The expression of AEP was higher in gastric cancer than that in the normal gastric mucosa, and AEP was an independent prognostic factor of gastric cancer [15–16]. However, the mechanism by which AEP promotes invasion and metastasis in gastric cancer remains unclear. AEP could control extracellular matrix renewal by degrading the fibronectin of the proximal renal tubule, thus, AEP overexpression may promote the proliferation and metastasis of tumors [17]. It has been reported that diffuse type gastric cancer is prone to get peritoneal metastasis, and tumor cells changed from epithelioid to mesenchymal-like cells in shape and function, then dropped, imbedded and located in peritoneal epithelium, finally became peritoneal metastatic lesion [5]. Thus, we speculated that AEP promote the invasion and metastasis of gastric cancer via EMT. In this study, we also utilized the antibody array to screen the relative signaling pathway that was correlated with AEP, and would provide some evidence for further research.

## RESULTS

### Higher expression of AEP in diffuse type gastric cancer than that in intestinal type gastric cancer

**Figure 1:**
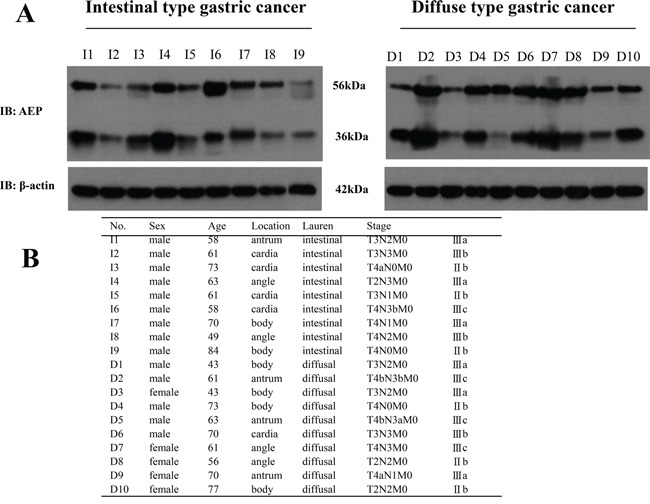
The expression of AEP was higher in diffuse type gastric cancer than that in intestinal type gastric cancer **A.** AEP expression in intestinal type gastric cancer and diffuse type gastric cancer were detected by western blot (Representation: I, intestinal type gastric cancer; D, diffuse type gastric cancer, *P*=0.032). **B.** Patients' basic characteristics.

We used western-blot to detect AEP expression in 9 patients with intestinal type gastric cancer and 10 patients with diffuse type gastric cancer. There were 14 male and 5 female patients and the median age was 61 years old. Patients' basic characteristics were listed in Figure 1. Proteins were obtained from fresh gastric cancer tissues after radical gastrectomy in our hospital. The results showed that the expression of AEP was much higher in diffuse type gastric cancer than that in intestinal type gastric cancer (seen in Figure 1, *P*=0.032).

### Higher expression of AEP, and lower expression of E-cadherin in peritoneal metastatic loci than that in primary gastric cancer

**Figure 2:**
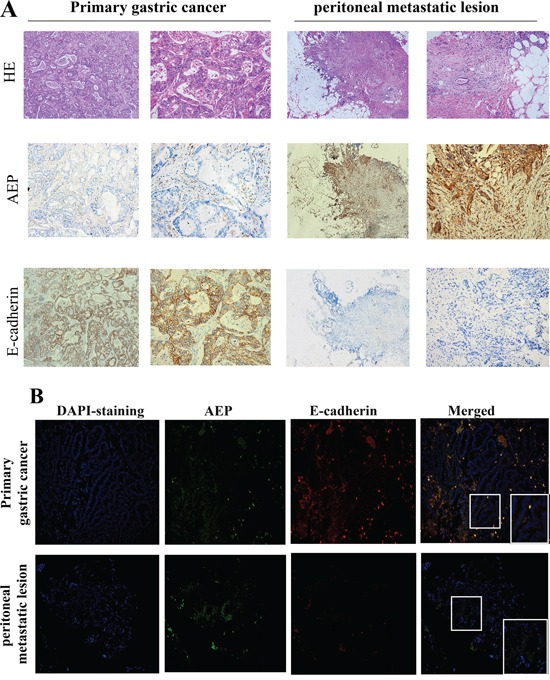
AEP and E-cadherin expression were detected both in primary gastric cancer and peritoneal metastatic loci **A.** The expression of AEP and E-cadherin in primary gastric cancer and metastatic peritoneal loci were firstly investigated by immunohistochemistry. AEP expression were higher and E-cadherin expression was lower in peritoneal metastatic lesions than in primary gastric cancer (magnified 50× in column 1,3; magnified 200× in column 2,4). **B.** The expression of AEP and E-cadherin in primary gastric cancer and metastatic peritoneal loci were investigated by immunofluorescence assay (magnified 200×). AEP, the secondary antibody of which were labeled with green fluorescence, was more strongly expressed in peritoneal metastatic lesions than in primary gastric cancer. E-cadherin, the secondary antibody of which was labeled with red fluorescence, was expressed in primary gastric cancer and could not be identified in peritoneal metastatic loci.

The expression of AEP and E-cadherin were detected with the immunohistochemistry method both in primary gastric cancer and the peritoneal metastatic loci of 30 patients simultaneously. AEP was found in 60% of primary gastric cancer and 100% of peritoneal metastatic loci. The IHC scores were 1.87 ±2.05 and 5.70 ±2.56 (*P*<0.01) in primary gastric cancer and peritoneal metastatic loci, respectively. E-cadherin was found in 63.33% of primary gastric cancer and 33.33% of peritoneal metastatic loci. The IHC scores were 3.52±3.49 and 1.53±2.75 (*P*<0.05) in primary gastric cancer and peritoneal metastatic loci. E-cadherin was negative both in primary and metastatic loci in eight patients (26.67%). (Figure 2A).

We also performed an immunofluorescence microscopic assay to detect the expression of AEP and E-cadherin in primary gastric cancer and peritoneal metastatic loci. The results showed that the expression of AEP (labeled with green fluorescence) was higher in peritoneal metastatic loci than that in primary gastric cancer and E-cadherin (labeled with red fluorescence) expression was lower in peritoneal metastatic loci than that in primary gastric cancer. (Figure 2B).

### Stably knocked-down or overexpressed AEP in SGC7901 and MKN45 gastric cancer cell lines and the proliferative ability was changed

**Figure 3:**
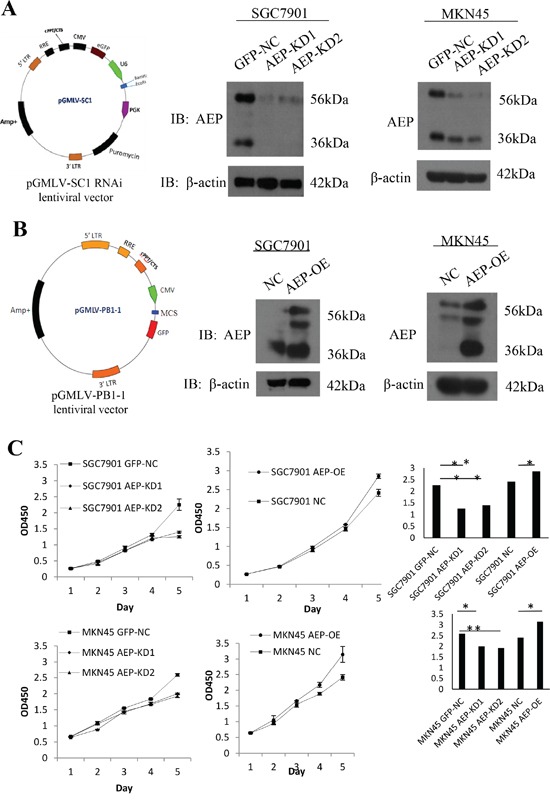
AEP knockdown and overexpressive vectors were constructed and stably transfected gastric cancer cell lines **A.** Constructing of the AEP knocked-down lentiviral vector, it was verified by western blot that AEP was inhibited corresponding to AEP knockdown. **B.** Overexpressing AEP lentiviral vector was constructed and AEP indeed increased corresponding to AEP overexpression. **C.** The proliferative curve in response to AEP overexpression or knockdown as evidenced by the CCK8 method and quantification of proliferative rate. *P<0.05, **P<0.01. (GFP-NC: control of empty vector; AEP-OE: AEP-overexpression; NC: negatively control; AEP-KD1: AEP-knocking down 1; AEP-KD2: AEP-knocking down 2)

To explore the role of AEP in gastric cancer, we stably overexpressed and knocked down AEP in SGC7901 and MKN45 cells (Figure 3A, 3B). We investigated the effect of AEP on the proliferation of SGC7901 and MKN45 cells using the CCK8 method. The result showed that knocking-down AEP markedly decreased proliferation, while the overexpressing AEP promoted gastric cancer cells' proliferative ability (Figure 3C, 3D). The quantitative data were listed in supplementary Table 2.

### The cellular morphology and the genes associated with EMT changed according to AEP overexpression or knockdown

**Figure 4:**
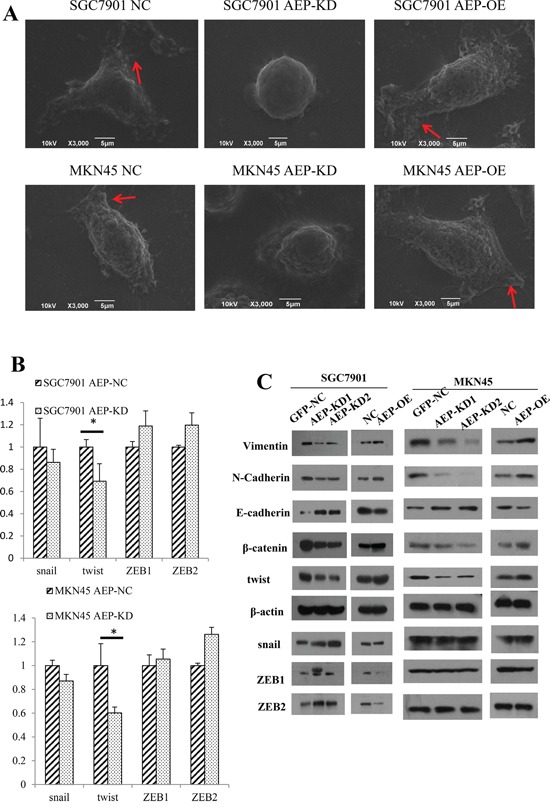
The morphologic change and expression of genes associated with EMT **A.** When AEP was knocked down, the cells got shrinkage and the number of microvilli and pseudopodia, as the red arrow indicated, decreased, and *vice versa*. **B.** The expression of snail, twist and ZEB1/2 as detected by real-time PCR. AEP knockdown inhibited the expression of twist significantly, but did not affect the mRNA level of snail and ZEB1/2. **C.** The changes in protein markers associated with EMT were detected with western blot. The epithelial marker E-cadherin increased and the mesenchymal markers N-cadherin, β-catenin and Vimentin decreased in response to AEP knockdown. Opposite results were obtained when AEP was overexpressed. Twist, but not snail and ZEB1/2, was significantly inhibited at protein level if AEP was knocked down.

Firstly, we observed the cell morphological changes under scanning electron microscope. When AEP was up-regulated, we found that the gastric cancer cells' morphology was stretched, and the quantity and length of microvilli and pseudopodia increased significantly. If AEP was knocked down, the number of microvilli and pseudopodia of gastric cancer cells decreased, and the cells trended to get tactful (Figure 4A).

Then, we detected the expression of transcriptional factors, such as snail, twist, ZEB1 and ZEB2 in gastric cancer cells using real-time PCR. The expression level of twist decreased in response to AEP knockdown (*P*<0.05). Although the expression of snail was also decreased, it wasn't obviously different (*P*>0.05). Nevertheless, AEP knockdown did not affect the expression of ZEB1 and ZEB2 at mRNA level (Figure 4B).

Finally, we detected the EMT relevant genes at protein level using western blot. When AEP was knocked down, the epithelial EMT marker E-cadherin increased, while the mesenchymal markers N-cadherin, β-catenin and Vimentin significantly decreased. The overexpression of AEP yielded the opposite results. We also investigated the expression of snail, twist, ZEB1 and ZEB2, the results showed that only twist was suppressed significantly when AEP was knocked down, the expression of snail, ZEB1 and ZEB2 didn't change. (Figure 4C).

These results suggested that AEP was associated with EMT in gastric cancer, but not all relevant transcriptional factors were involved in this association. We inferred that AEP might promote EMT via some special signaling pathway.

### Regulating AEP can change the invasive and metastatic ability of gastric cancer cells, and cell cycle arrest by AEP knockdown

**Figure 5:**
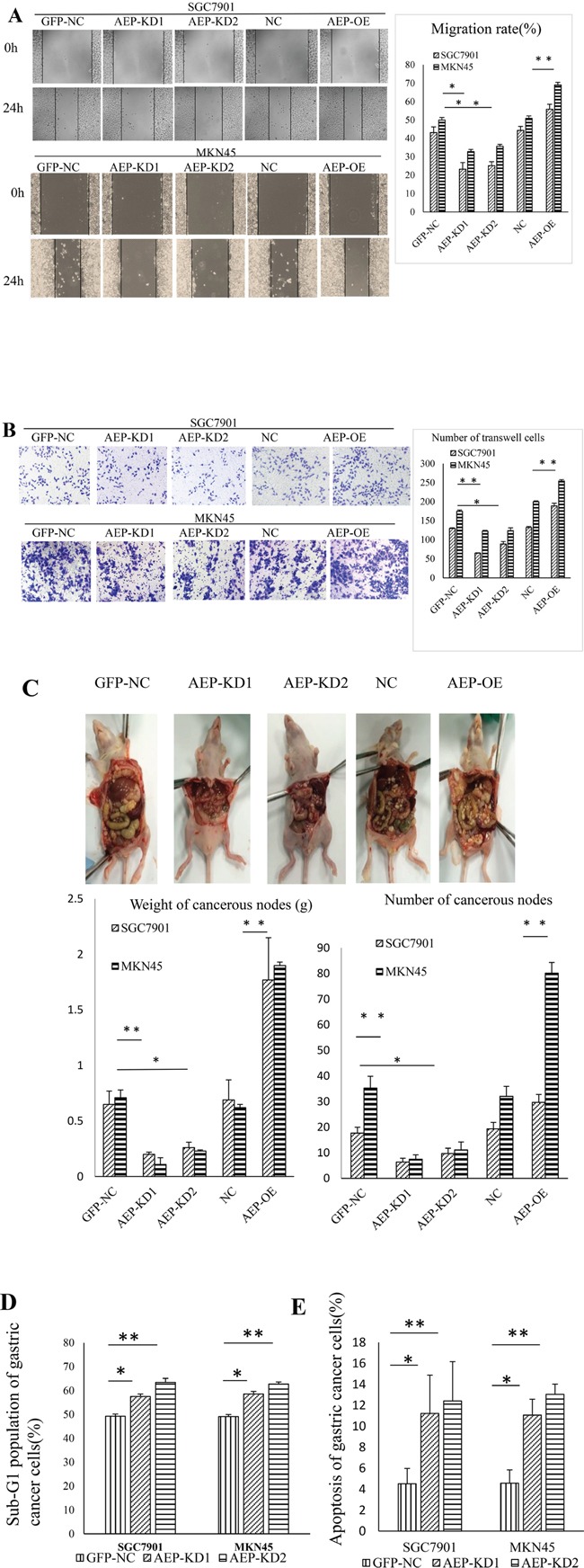
The changes of function in gastric cancer cells when AEP was knocked-down or overexpressive **A.** The change in cell migration in response to AEP overexpression or knockdown as evidenced by the scratch method. The migratory ability decreased in response to AEP inhibition and *vice versa*. **B.** The change in invasiveness as evidenced by the transwell method. The invasive ability decreased in response to AEP inhibition and *vice versa*. **C.** The change of cancerous nodes in peritoneal cavity in response to AEP knockdown and overexpression in nude mice. AEP inhibition decreased the number and the weight of tumor nodules in peritoneal cavity, and *vice versa*. The comparison of the weight and the number of cancerous nodes in peritoneal cavity was also shown in this figure. **D.** The cell cycle arrested at G1 stage if AEP was knocked down. The population of sub-G1 cells significantly increased. **E.** After PI staining, the percent of apoptotic cells increased when AEP was knocked-down. *P<0.05, **P<0.01. (GFP-NC: control of empty vector; AEP-OE: AEP-overexpression; NC: negatively control; AEP-KD1: AEP-knocking down 1; AEP-KD2: AEP-knocking down 2)

We also performed scratch and transwell assays to assess the horizontal and vertical migratory abilities of SGC7901 and MKN45 cells. The results confirmed that knocking-down AEP can inhibit the migratory and invasive abilities of gastric cancer cells, and *vice versa* (Figure 5A, 5B). AEP was overexpressed or knocked down in gastric cancer cells, and these cells were injected into the peritoneal cavity of nude mice. The group of AEP knockdown was found that the number of tumor nodules in peritoneal cavity decreased. While injecting cells of AEP overexpression, the cancerous nodes increased in peritoneal cavity of nude mice (Figure 5C). We also investigated the change of apoptosis and cell cycle through flow cytometry if AEP was knocked down. The percent of apoptotic cells after propidium iodide (PI) staining and the population of sub-G1 cells increased significantly in AEP knockdown gastric cancer cells than those in scramble-treated cells (Figure 5D and 5E). The quantitative data were seen in supplementary Table 2.

### AEP promotes invasion and metastasis by regulating multiple protein involving some important signaling pathways

**Figure 6:**
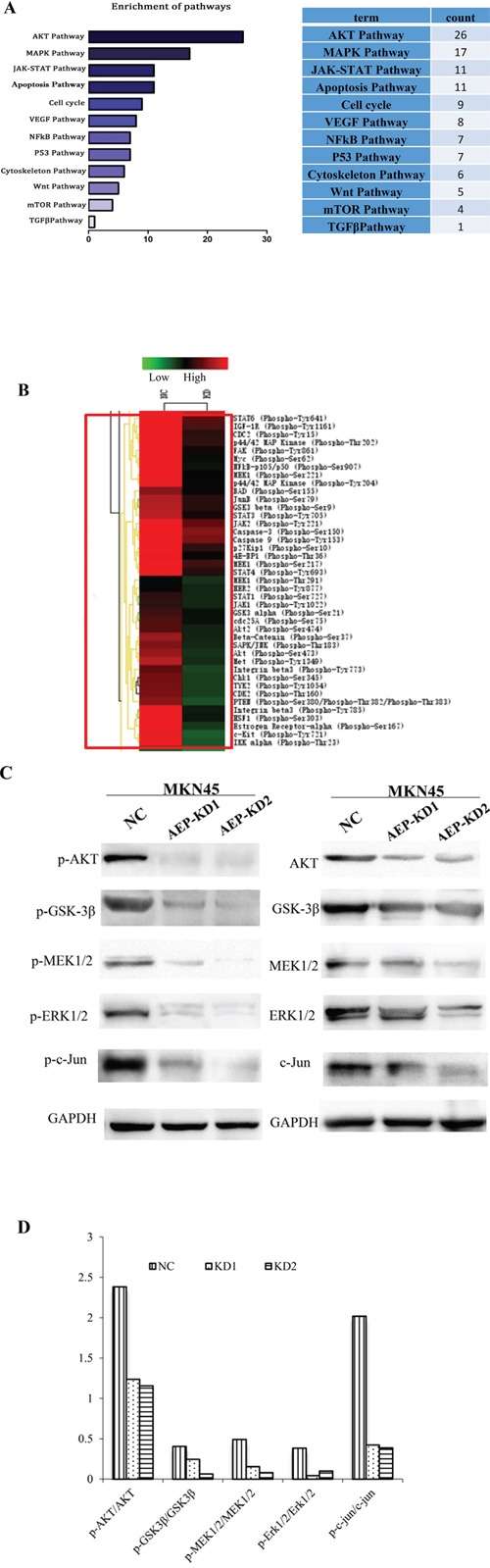
The analysis of the differential expressive phosphorylation proteins by microarray **A.** The canonical signaling pathways regulated by AEP based on the enrichment analysis using DAVID software. **B.** The phosphorylation ratio was used as the modulating difference of phosphorylation sites between MKN45 gastric cancer cells and AEP knocked-down MKN45 cells. The top twenty decreased phosphorylation proteins were listed in this figure. **C.** The expression of phospho-AKT/AKT, phospho-GSK-3β/GSK-3β in AKT signaling pathway, and phospho-MEK1/2/MEK1/2, phospho-ERK1/2/ERK1/2, phospho-c-Jun/c-Jun in MAPK signaling pathway, both in AEP knockdown group and control group. **D.** The ratio of phosphorylation proteins/total proteins. The quantitative analysis indicated that the ratio in AEP knockdown group were much lower than that in AEP control group (all of the *P* values <0.05).

To understand functional relationships and mechanisms of differential alteration in protein phosphorylation in response to AEP knockdown and to derive probable AEP-modulated signaling pathways in gastric cancer cells, we used phosphorylated antibody chip. The analyzed antibody microarray contained 269 different antibodies representing markers for 12 biological pathways including apoptosis, cell cycle, signal transduction, cytoskeleton, and so on. It was analyzed that the phosphorylation level of AKT and MAPK signaling pathways decreased significantly among the 12 different networks (Figure 6A). We identified a spectrum of proteins whose phosphorylation levels decreased more than 15% in AEP knockdown gastric cancer cells. The phosphorylation levels at 92 sites were reduced more than 15%. By cluster analysis, the top twenty down-regulated phosphorylation proteins were listed in Figure 6B.

Based on the results of clustering analysis, we detected the expression of the phosphorylation sites mainly in AKT and MAPK signaling pathways through western-blot. We investigated the expression of phospho-AKT, phospho-GSK-3β in AKT signaling pathway, and phospho-MEK1/2, phospho-ERK1/2, phospho-c-Jun in MAPK signaling pathway, even the total protein expression of AKT, GSK-3β, MEK1/2, ERK1/2, and c-Jun. The results showed that these phosphorylated genes' expression were inhibited significantly, even though the total protein expression also slightly decreased, when AEP was knocked down (Figure 6C). The ratio of phosphorylation/total proteins decreased significantly when AEP was knocked down, comparing with the control group (*P*<0.05) (Figure 6D). Therefore, AEP knockdown could prevent the invasion and metastasis of gastric cancer mainly by inhibiting the activity of the phosphorylation sites in AKT and MAPK signaling pathways.

## DISCUSSION

Asparaginyl endopeptidase is a newly identified lysosomal protease of the C13 family of peptidases with strict specificity for asparagine bond cleavage [18]. AEP has recently garnered the attention of researchers because several studies have verified that AEP is highly expressed in tumors but not in normal tissues. Moreover, high AEP expression correlated with a poor prognosis and short survival time in several cancers, such as in breast, colorectal, prostate and ovarian cancer [19–22]. Several functions have been reported for AEP, including the processing and presentation of antigens [23], modulation of fibronectin degradation [17], promotion of angiogenesis factor release, activation of matrix metalloproteinases [24–25], and participation in tumor-associated macrophage function [26]. Therefore, AEP is closely associated with the development and metastasis of cancer, and AEP has become a new hot spot in tumor treatment, cell immunity, diagnosis and prognostic evaluation. There were two papers about AEP in gastric cancer that have been published. AEP was higher expressive in low-differentiated gastric cancer cell lines than that in high- and middle- differentiated gastric cancer cell lines. Two papers concordantly verified that a high expression of AEP correlated with a poor prognosis and that AEP was an independent prognostic factor of overall survival in gastric cancer [15–16]. However, the mechanism by which AEP promotes the invasion and metastasis of gastric cancer remains elusive.

In this study, firstly we found that AEP was higher expressive in diffuse type gastric cancer than that in intestinal type gastric cancer, then we detected the expression of AEP and E-cadherin both in primary gastric cancer and peritoneal metastatic loci samples simultaneously. We found that the expression of AEP was much higher in peritoneal metastatic loci than in primary gastric cancer. In primary gastric cancer, the positive rate of AEP expression was similar to that reported in previously published papers [15–16]. However, the expression of E-cadherin was much lower in peritoneal metastatic loci than in primary gastric cancer. E-cadherin is an important protein marker of EMT. Therefore, we inferred that AEP might promote invasion and metastasis by inducing EMT in gastric cancer. Subsequently, we generated constructs to overexpress AEP or lentivirally knock down AEP in the gastric cancer cell line SGC7901 and MKN45. AEP knockdown was found to inhibit proliferation, invasion and metastasis *in vitro* and *in vivo*, and the cell cycle was blocked at G1 stage.

EMT is a significant event during tumor invasion and metastasis. EMT in tumor cells is characterized by changes in biomarkers, such as cell membrane proteins, cytoskeletal proteins, extracellular matrix proteins and some transcription factors, in addition to changes in the cellular morphology, cell polarity and ability to migrate and invade. E-cadherin is inhibited [27–28] and N-cadherin is activated during EMT in gastric cancer [29–30], and the expression levels of the cytoskeletal protein markers β-catenin and Vimentin are also increased [31–32]. Some transcriptional factors that are relevant to EMT are activated, such as snail, twist, ZEB1 and ZEB2 [33–34].

If AEP was knocked-down, the number of microvilla and pseudopodia decreased and cells shrinked. We found that E-cadherin expression was increased when AEP was knocked down, and N-cadherin, β-catenin and Vimentin, which are mesenchymal protein markers of EMT, decreased in response to AEP knockdown, and *vice versa* if AEP was overexpressed. Interestingly, AEP knockdown did not suppress all transcriptional factors at the mRNA level. The expression of snail and twist were inhibited, but ZEB1 and ZEB2 expression did not markedly change. Therefore, we speculated that the modulation of EMT by AEP may be associated with some unique signaling pathway.

Post-translational modification of proteins, such as the phosphorylation of serine-, threonine- and tyrosine- residues, can initiate multiple down-stream signaling events, causing protein-protein interaction, subsequently activate signaling cascades, leading to cell abnormal proliferation and differentiation. In order to investigate the mechanism of AEP promoting gastric cancer invasion and metastasis, we utilized the phosphorylated antibody microarray to detect which genes would be inhibited if AEP was knocked-down, even the associated signaling pathways. The results showed that much more phosphorylation sites in AKT and MAPK signaling pathways were inhibited among the 12 signaling pathways. AKT is a serine/threonine kinase activated downstream of integrin, which is a receptor for various proliferation and bioactive substances as well as the extracellular matrix receptor. AKT activation or overexpression can serve as a biomarker for predicting the metastasis of human gastrointestinal cancer [35]. The mitogen-activated protein kinase (MAPK) signaling pathway is widely expressed in multicellular organisms, with critical roles in multiple biological processes, such as cell proliferation, migration, and invasion. The function of the three central kinases of the MAPK signaling pathway, ERK, JNK and p38 were all involved in the metastasis and invasion of gastric cancer [36–39].

Since the AKT and MAPK signaling pathways are well-known to play an essential role in regulating cell proliferation and metastasis in gastric cancer, we detected the genes expression of phospho-Akt/Akt, phospho-GSK-3β/GSK-3β, phospho-MEK1/2/MEK1/2, phospho-Erk1/2/Erk1/2and phospho-c-Jun/c-Jun at protein level, which are key genes in AKT and MAPK signaling pathways. We found that the ratio of phosphorylated proteins/total proteins in AKT and MAPK signaling pathways decreased significantly when AEP was knocked-down. Therefore, AEP knocking-down mainly inhibited the activation of phosphorylated proteins in AKT and MAPK signaling pathways, which indicates a key role of AKT and MAPK pathways in regulating AEP-induced invasion and metastasis in gastric cancer.

Overall, this study verified that AEP could promote invasion and metastasis by modulating EMT in gastric cancer, especially in diffuse type gastric cancer. This effect mainly involved AKT and MAPK pathways, which deserves further study. Therefore, AEP may serve as a new diagnostic and therapeutic target for gastric cancer with peritoneal metastases.

## MATERIALS AND METHODS

### AEP and E-cadherin in primary and metastatic loci of gastric cancer

Paraffin-embedded samples of primary gastric cancer and peritoneal metastatic loci were collected from patients at Shanghai Zhongshan Hospital, which is affiliated with Fudan University, after obtaining the patients' informed consent and institutional review board approval. We used immunohistochemistry to investigate the expression of AEP and E-cadherin in the samples of surgery or endoscopy. The staining intensity was graded as follows: weak staining, moderate staining and strong staining, which were scored from 1 to 3. The staining square was graded by the presence of positively stained tumor cells: 0-10% tumor cells, 10% to 50% tumor cells, and >50% tumor cells, which were also scored from 1 to 3. The immunohistochemical value was acquired as the score of staining intensity multiplied the score of staining square [40].

### Cells and animals

The gastric cancer cell lines of SGC7901 and MKN45 were maintained in RPMI 1640 containing 10% FBS. The animal care and experimental protocols were conducted in accordance with the guidelines established by the Shanghai Medical Experimental Animal Care Commission. Male athymic BAL B/C nude mice were purchased from the Shanghai Institute of Material Medicine, Chinese Academy of Science, and were raised under specific pathogen-free conditions. 2×10^6^ cells were injected into the abdominal cavity to mimic a peritoneal metastasis, the mice were sacrificed after 4 weeks. Each group included eight mice.

### Antibodies

The primary antibodies were obtained from the following sources: AEP (R&D, #AF2199), Vimentin (Abcam, ab28028), twist (Abcam, ab49254), snail (Abcam, ab53519), E-cadherin (Abcam, ab15148), N-cadherin (Abcam, ab18203),β-catenin (Abcam, ab16051), ZEB1 (Genetex, GTX109031), ZEB2 (Genetex, GTX85180) and HRP-conjugated beta actin monoclonal antibody (Proteintech, HRP-60008). Antibodies of Phospho-Akt/Akt, phospho-GSK-3β/GSK-3β, phospho-MEK1/2/MEK1/2, phosphor-Erk1/2/Erk1/2, and phosphor-c-Jun/c-Jun, all purchased from Cell Signaling Technology. The fluorescent secondary antibodies were obtained from the following sources (MultiSciences Biotech Co., Ltd.): DyLight™488 conjugated rabbit anti-goat IgG (H+L), Goat anti-mouse IgG (H+L chain specific).

### Recombinant plasmid construction, lentivirus production and transduction

① Knocking-down AEP: A recombinant lentiviral plasmid encoding AEP open reading frame(ORF) was constructed with a Flag tag in C terminal as previously described [41]. In the meantime, two shRNA expression cassettes specifically targeting AEP gene was constructed using for AEP knockdown. The sequences of the shRNA oligonucleotides were listed at Supplementary Table S1. All of the recombinant plasmids carried a puromycin-resistant gene. After validated by sequencing, recombinant plasmids were co-transfected with lentivirus packaging helper plasmids (psPAX and pMD2.G) into HEK293T cells. SGC7901 and MKN45 cells were transduced with the lentivirus. (Figure 3A). ② Overexpression of AEP: A human AEP expression plasmid was constructed. The plasmid was cloned into the lentivirus system using the pGMLV-PB1-1 vector, then was stably transducted into gastric cancer cell lines (Figure 3B).

### Measurement of cellular behavior

We observed the change of cellular morphology with scanning electron microscopy (JEOL6390LV). The cellular behavior, including proliferation and cell motility, was measured using the CCK8 method, cell scratch assay and transwell test. Briefly, cells were cultured in a 24-well plate at a density of approximately 2.5×10^4^ cells/well. For the scratch assay, a line was scratched into a confluent monolayer of cells to investigate cell motility. We used a transwell chamber to observe the invasiveness of cells. The cells that had invaded the Matrigel were fixed for 5 min in 10% glutaraldehyde and stained with hematoxylin for 5 min, then were counted under a microscope. The percent of the different phases of the cell cycle were determined by flow cytometry as previously described [42]. Every experiment was repeated three times.

### Measurement of EMT markers

① Real-time PCR was used to measure the expression of EMT markers at the mRNA level. Total RNA was extracted from cultured cells using TRIzol® reagent (Invitrogen), and then was reversely transcribed to generate cDNA. The PCR amplification of cDNA was monitored in real time using the primer pairs shown as below, snail: F:5′-TGCCCTCAAGATGCACATCCGA-3′,

R:5′-GGGACAGGAGAAGGGCTTCTC-3′;twist: F:5′-GCCAGGTACATCGACTTCCTCT-3′,

R:5′-TCCATCCTCCAGACCGAGAAGG-3′;ZEB1: F:5′-GGCATACACCTACTCAACTACGG

-3′, R:5′-TGGGCGGTGTAGAATCAGAGTC-3′; ZEB2:F:5′-AATGCACAGAGTGTGGCAAG

GC-3′, R:5′-CTGCTGATGTGCGAACTGTAGG-3′. ② The changes in protein expression were investigated by western blot. Samples containing 30μg of total protein were separated by SDS-PAGE on a 10% acrylamide gel. The separated polypeptides were transferred to nitrocellulose membranes, probed with antibodies and visualized with enhanced chemiluminescence, as previously described [43].

### Phosphoprotein profiling by the Phospho Explorer antibody microarray

The Phospho Explorer antibody microarray CSP100, which was designed and manufactured by (Fullmoon Biosystems Inc., Sunnyvale, CA, USA), contains 269 antibodies. Each of the antibodies has two replicates that are printed on coated glass slide, along with multiple positive and negative controls. Cell lysates obtained from MKN45 and MKN45 AEP-KD cell lines were biotinylated with Antibody Array Assay Kit. The slides were scanned on a GenePix 4000 scanner and the images were analyzed with GenePix Pro 6.0 (Molecular Devices, Sunnyvale, CA).

### Statistical analysis

Two-tailed Student's *t* test was used to analyze differences between the protein overexpression and knockdown groups from *in vitro* and *in vivo* experiments. One-way analysis of variance was initially performed to identify overall significant changes before using two-tailed paired or unpaired Student's *t* test. A two-tailed value of *P*<0.05 was used to indicate a significant difference.

## SUPPLEMENTARY TABLES


