# Supplementary material for: Asparaginyl endopeptidase promotes the invasion and metastasis of gastric cancer through modulating epithelial-to-mesenchymal transition
Source: Oncotarget. 2016 Apr 20;7(23):34356–70. doi: 10.18632/oncotarget.8879 (PMC5085161; doi:10.18632/oncotarget.8879)
Supplement: Supplementary file 1 [file oncotarget-07-34356-s001.pdf]

Asparaginyl endopeptidase promotes the invasion and metastasis of gastric cancer through modulating epithelial-to-mesenchymal transition

SUPPLEMENTARY TABLES

Supplementary Table S1: The AEP amplification primer sequence and AEP shRNA oligonucleotides sequences

	AEP shRNA oligonucleotides sequences
	5' to 3'
AEP-shRNA-1-top	gatccGATGGTGTTCTACATTGAATTCAAGAGATTCAATGTAGAA CACCATCTTTTTTg
AEP-shRNA-1-bottom	aattcAAAAAAGATGGTGTTCTACATTGAATCTCTTGAATTCAAT GTAGAACACCATCg
AEP-shRNA-2-top	gatccAAACTGATGAACACCAATGATTTCAAGAGAATCATTGGT GTTCATCAGTTTTTTTTTg
AEP-shRNA-2-bottom	aattcAAAAAAAAACTGATGAACACCAATGATTCTCTTGAAATCATT GGTGTTCATCAGTTTg
	AEP ORF expression primer sequences
	5' to 3'
human AEP-F(XhoI+Flag)	CCGCTCGAGGCCACCATGGACTACAAGGACGATGACGACAA GGTTTGGAAAGTAGCTG
human AEP-R(EcoR I)	CCGGAATTCTCAGTAGTGACCAAGGCACA

Supplementary Table S2.

The detailed data of experiments in SGC7901 gastric cancer cells *in vitro* and *in vivo*

SGC7901	NC	OE	GFP-NC	KD1	KD2
CCK8-Day1	0.2675±0.0014	0.2676±0.0016	0.2655±0.0134	0.2646±0.0293	0.2643±0.006
Day3	0.905±0.0457	0.977±0.0287	0.9±0.062	0.8215±0.0054	0.8205±0.0183
Day5	2.4139±0.093	2.8535±0.059*	2.2535±0.178	1.2547±0.0373**	1.3995±0.0241**
Migration(%)	44.35±2.1121	55.83±2.6616**	43.14±3.1032	23.23±3.6*	25.16±2.1517**
Transwell	132±2.83	189±7.07**	130±1.41	65±0.71**	89±6.36*
Number	19.33±2.52	29.67±3.06**	17.67±2.31	6.33±1.53**	9.67±2.08*
Weight(g)	0.69±0.18	1.77±0.38**	0.65±0.12	0.2±0.02**	0.26±0.05*

*$P<0.05$, **$P<0.01$

The detailed data of experiments in MKN45 gastric cancer cells *in vitro* and *in vivo*

MKN45	NC	OE	GFP-NC	KD1	KD2
CCK8-Day1	0.6424±0.0145	0.647±0.0239	0.6655±0.009	0.6595±0.0024	0.6405±0.0015
Day3	1.5332±0.073	1.6651±0.0042	1.5493±0.0078	1.4166±0.0486	1.4493±0.0187
Day5	2.4096±0.0871	3.1465±0.2515*	2.5851±0.0383	1.9999±0.0193*	1.9253±0.0441**
Migration(%)	50.91±1.2856	69±1.4142**	49.97±1.3786	32.82±1.1571*	35.71±1.0101**
Transwell	199±2.444	254±3.778**	174±3.556	122±1.778**	124±2.333*
Number	32.01±3.91	80.18±4.13**	35.25±4.61	7.46±1.68**	11.13±3.1*
Weight(g)	0.62±0.03	1.9±0.03**	0.71±0.07	0.11±0.06**	0.23±0.01*

*$P<0.05$, **$P<0.01$

The change of apoptosis and cell cycle if AEP was knocked down

	SGC7901			MKN45		
	GFP-NC	AEP-KD1	AEP-KD2	GFP-NC	AEP-KD1	AEP-KD2
Sub-G1 population(%)	48.42±1.10	56.39±1.09*	62.58±0.61**	48.33±0.79	57.61±1.12*	62.30±0.89**
Apoptotic Cells(%)	4.52±1.46	11.22±3.65*	12.41±3.74**	4.58±1.26	11.06±1.51*	13.05±0.96**

*$P<0.05$, **$P<0.01$
